# Modeling the development of the post-natal mouse thymus in the absence of bone marrow progenitors

**DOI:** 10.1038/srep36159

**Published:** 2016-11-08

**Authors:** Daniela Zaharie, Radu D. Moleriu, Felix A. Mic

**Affiliations:** 1 Faculty of Mathematics and Computer Science, West University of Timisoara, 4 Vasile Parvan Blvd, Timisoara, Romania; 2 Department of Functional Sciences, “Victor Babes” University of Medicine and Pharmacy, 2 Eftimie Murgu Sq., Timisoara, Romania

## Abstract

Many mathematical models have been published with the purpose of explaining aspects of T-cell development in the thymus. In this manuscript we adapted a four-compartment model of the thymus and used a range of mathematical approaches with the aim of explaining the dynamics of the four main thymocyte populations in the mouse thymus, from the emergence of the first fetal thymocyte until the death of the animal. At various pre-natal and post-natal stages we investigated experimentally the number and composition of thymocytes populations, their apoptosis and proliferation, along with data from literature, to create and validate the model. In our model the proliferation processes are characterized by decreasing proliferation rates, which allows us to model the natural involution of the thymus. The best results were obtained when different sets of parameters were used for the fetal and post-natal periods, suggesting that birth may induce a discontinuity in the modeled processes. Our model is able to model the development of both pre-natal and post-natal thymocyte populations. Also, our findings showed that the post-natal thymus is able to develop in the absence of the daily input of bone marrow progenitors, providing more evidence to support the autonomous development of the post-natal thymus.

The emergence of the embryonic mammalian thymus, its subsequent rapid development in the early stages of post-natal life and then its gradual involution later in life are complex processes that are incompletely understood^1^. Thymocyte proliferation, differentiation, death, and bi-directional interactions with the thymus stromal component are all complex processes that occur simultaneously in the organ. Although all thymocytes from the thymus are mixed together without apparent physical boundaries, they can be divided into specific populations based on the expression of certain markers. In case of mouse thymus, based on the expression of CD4 and CD8 markers^2^, the thymocytes can be divided into four main populations: CD4−CD8− double negative (DN, N(t)), CD4+CD8+ double positive (DP, P(t)), mature CD4+CD8− single positive (SP4, SP4(t)), and mature CD4−CD8+ single positive (SP8, SP8(t)). The current view of thymocyte developmental flow in the thymus indicates that bone marrow progenitors enter the thymus on a daily basis where they first generate DN thymocytes that subsequently turn into DP ones that undergo intense proliferation, differentiation and apoptosis as they generate the mature SP4/SP8 thymocytes, which are exported as functional T-cells from the thymus.

The development of the fetal mouse thymus begins at mid-gestation when the thymus bud, an area around the third pharyngeal pouch^3^, is colonized around E10.5–11 stage by hematopoietic progenitors originating in the fetal liver^4^,^5^, which are transported through the blood stream^6^. Soon afterwards, the first DN thymocytes appear in the thymus primordium, at around E11 of gestation^7^. In the next few days the DN thymocytes proliferate intensively and form a sizable population by the stage E14. The first DP thymocytes can be detected experimentally around E15 of pregnancy^8^,^9^. Shortly afterwards, at E16 stage, the SP4 and then the SP8 thymocytes can be detected experimentally in the embryonic thymus^8^,^9^. At birth, the fetal thymus contains the four main thymocyte populations which are found in the post-natal thymus. In the first few weeks after birth, the cellularity of the mouse thymus expands rapidly, reaching a plateau at 4–6 weeks of age and gradually involutes afterwards, although the organ continues to operate in old age^10^.

There are functional differences between the pre-natal and post-natal thymuses. Relative to the adult thymus, the neonatal thymus has greater thymocyte proliferation and a predominance of immature thymocytes^11^. The proliferation of the DN and DP thymocyte populations is higher in the fetal thymus than in the post-natal one^12^. When the proliferation of the four main thymocyte populations was examined in the 5 week old mouse thymus, the results showed that 90% of all proliferating thymocytes are DPs and most of the remaining ones are DNs^13^. A small percentage of the mature SP4 and SP8 thymocytes still proliferate after selection, in both the neonatal^14^, and the adult mouse^15^. Data on the dynamics of thymocyte apoptosis in the adult mouse thymus^16^ and the composition of the four main thymocyte populations in the pre-natal thymus at several stages^17^,^18^ were obtained from the literature. Based on experimental observations, the change over time in the number of precursors in the pre-natal thymus appears to follow a logistic type of growth^19^.

For a long time it was accepted that the thymus does not contain any self-renewal capacity and that its thymopoiesis required a constant input of bone marrow progenitors. In spite of a considerable amount of research being devoted to the isolation and characterization of these progenitors, their molecular signature was not clearly identified in the adult mouse^20^,^21^,^22^. There are experimental observations which support this hypothesis, such as thymus transplants, in which resident thymocytes generate a single wave of mature T cells, due to the fact that precursors originating from the bone marrow of the host rapidly replace the resident cells of the transplanted thymus^23^. When wild-type thymi are transplanted into immuno-deficient or Rag2-deficient hosts (which lack bone marrow precursors), the competent thymocyte populations from the graft are rapidly replaced by the incompetent precursors from the bone marrow of the host, and by 3 weeks after surgery, the export of mature T cells fails^24^,^25^. Nonetheless, recent results from multiple knock-out mouse experiments support the concept of a thymus that does not need a continuous inflow of bone marrow progenitors to maintain normal thymopoiesis^26^,^27^,^28^. Peaudecerf *et al*.^26^, found a population of thymocytes in the neonatal thymi transplanted into interleukin 7 receptor–deficient hosts which could self-renew and continuously generate mature thymocytes, resulting in the development of a full peripheral T cell repertoire in just one month after transplantation. In another approach^27^, a strain of triple knock-out mice that lack T, B, and natural killer cells, and are devoid of T-cell progenitors, showed sustained T-cell development that lasted for several months when transplanted with a wild-type thymus.

Molecular and cellular investigations provide meaningful, individual observations but from such static experiments it is difficult to obtain dynamic information. Mathematics provides the appropriate tools for simultaneously analyzing thymocyte dynamics and the propagation of a perturbation from a population to other thymocyte populations. Thymus functions and various aspects of thymocyte apoptosis, proliferation, selection and maturation were previously modeled by differential equations, cellular automata or agent-based models^29^,^30^,^31^,^32^,^33^. Most thymus compartmental models^29^,^30^,^31^ use density and/or competition based regulation of the population sizes, which are appropriate for modeling homeostasis, but cannot accurately describe natural involution of the organ. With the aim of modeling thymus development from the appearance of the first thymic progenitor until late life involution stages, we modified a traditional compartmental model^29^,^30^ by introducing exponentially decreasing proliferation rates in order to be able to model the involution of the organ. Besides this modification, the proposed mathematical model also incorporates a delay between the DN and DP compartments and contains a compartment which models the collection of the apoptotic cells that are being phagocytosed by the stromal cellular component of the thymus. We assumed that all dead thymocytes follow a similar death pathway and are treated equally by the thymic stroma, irrespective of their population of origin.

Thus we arrived at two main classes of mathematical models (both based on decreasing proliferation rates), one which does not incorporate (M1) and another one which incorporates density-based control of population growth (M2). Each of these two classes of models were further divided into two variants, which allow (V1) or do not allow (V2) different sets of parameters in the modeling of the pre-natal and post-natal thymocyte dynamics. The models were separated initially on the basis of their fitting quality, and the models with poorer fitting were not pursued further. Models with comparable fitting were examined for their ability to model the dynamics of the thymocyte populations and to match crucial developmental time points in the development of the fetal thymus, from our data or from the literature.

We modeled the development of the fetal and post-natal thymocyte populations, their proliferation and the dependence of the post-natal thymus on the inflow of progenitors from the bone marrow, on a time scale ranging from the emergence of the first fetal DN thymocyte until the death of the mouse, at approximately 2 years of age. We used our experimental data on pre-natal and post-natal thymocyte apoptosis, the numerical value and percentage of thymocyte populations at indicated pre-natal and post-natal stages^34^, and findings from the literature regarding the proliferation, apoptosis, and numerical changes in thymocyte populations in the fetal and post-natal thymus in order to develop and validate the model. We also examined the role and significance of the discontinuity in the proliferation rate of thymocytes during the birth period. We found that the proliferation rates of all four thymocyte populations underwent significant changes at birth time and that these changes correlated with experimental observations. Also, our model showed that the post-natal thymus is able to develop in the absence of the daily input of progenitors from the bone marrow, providing further evidence to support the autonomous development of the post-natal thymus.

## Results

### The fitting quality of the mathematical model variants and their ability to model the emergence of thymocyte populations in the pre-natal thymus

All four variants are able to model both the growth and involution phases of the thymus from the appearance of the first fetal DN thymocyte until the final stages of mouse life. The results reported in Table 1 suggest that by combining density based control of the growth with decreasing proliferation rate one can fit the experimental data, but that the quality of the fit is not better than that obtained using only decreasing proliferation rates (M1.V1 versus M2.V1). On the other hand, the constraint on non-discontinuity in the proliferation function at the moment of birth has a smaller impact on models which involve density based factors (M2.V1 and M2.V2).

To further investigate the fitting quality of the modeling, we examined the correspondence between the time points of the appearance of the DN thymocyte population in the fetal thymus as obtained using the simulations of all four models, and those reported in the literature^3^,^4^,^5^,^7^,^8^,^9^. The results are displayed in Table 2 and show that in the model M1.V1 the emergence of the fetal DN thymocytes appear to be in agreement with the experimental data^7^, while in M1.V2 they appear somewhat later but still before the E11 stage. In model M2 the moment of the first DN thymocytes emergence is also before the stage E11, in both variants, M2.V1 and M2.V2 (Table 2), but the fitting quality in both variants of this model is poorer (Table 1).

Based on the fitting quality and the match with the emergence of the DN thymocyte population in the fetal thymus, the best approach to modeling the dynamics of the thymocyte populations in both the fetal and the post-natal thymus is the one with a decreasing proliferation rate and birth discontinuity of rates (M1.V1).

### Modeling the development of the four main thymocyte populations in the pre-natal thymus in mice

**Figure 1:**
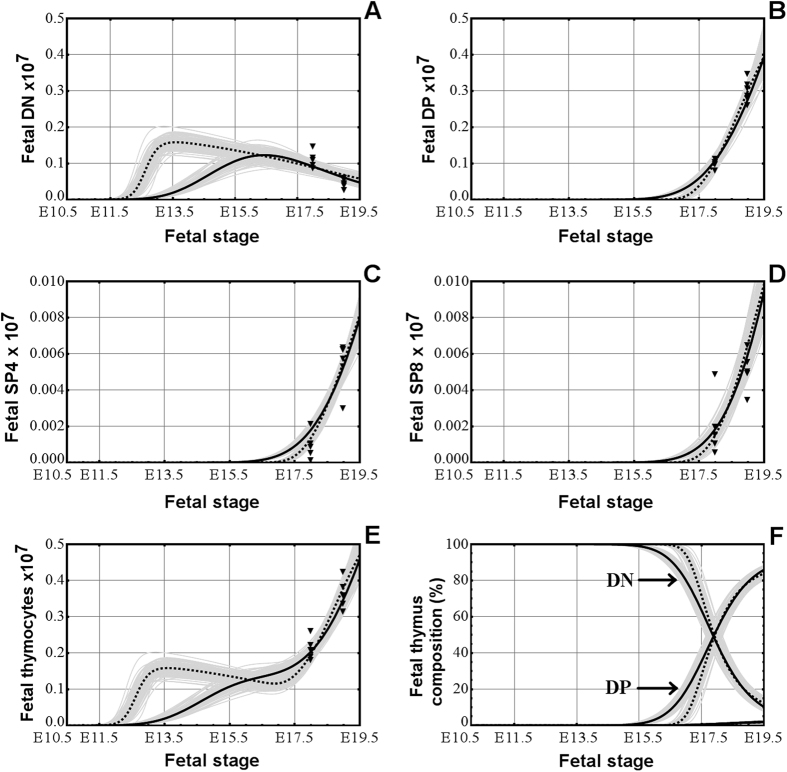
The development of the DN (**A**), DP (**B**), SP4 (**C**), and SP8 (**D**) thymocyte populations in the pre-natal thymus, simulated by model M1.V1 (continuous line) and model M2.V1 (dotted line). (**E**) The changes in the whole number of thymocytes in the pre-natal thymus in both models. (**F**) The dynamics of the thymocyte composition in the pre-natal thymus, in both models. The filled triangles on each chart are the experimental number of thymocytes at the respective time points.

Next we investigated the ability of the two selected variants of models (M1.V1 versus M2.V1 in Table 1) to match the emergence of DP, SP4, and SP8 thymocyte populations during the development of the fetal thymus and we plotted the simulated curves. In the model M1.V1 (Fig. 1A, continuous line, and Table 2), the first DN thymocytes appear between E10–10.5, and the DN population grows rapidly in the fetal period, peaking at E16.5, around the time when the DP population starts to increase significantly, and then declines until birth. The first DP thymocyte is generated shortly before E13.5 (Fig. 1B, continuous line, and Table 2), while the SP4 and SP8 thymocytes appeared just before E14.5 (Fig. 1C,D, continuous line, and Table 2), in agreement with the experimental findings^8^,^9^. There is also a delay of roughly 3.5 days between the emergence of DN and DP thymocytes, and about one day between the emergence of DP and SP4/SP8 thymocytes (columns SP4 and SP8 versus column DP, Table 2), also consistent with previously published data^8^,^9^. After the emergence of fetal DP, SP4 and SP8 thymocytes, their populations grew exponentially until birth. The model M1.V1 is able to match the onset of fetal DP, SP4, and SP8, and the delays between DN-DP, DP-SP4, and DP-SP8, as shown in Table 2.

When the model M2.V1 was used in a simulation we found several mismatches with the experimental results regarding the main time points in the fetal development of the thymus. In this model, although the DN thymocytes appear only a short time later than in the previous model, there is a very early peak just prior to E13 stage, caused by the higher initial value of the proliferation rate and then a slight decrease due to a dropping proliferation rate (Fig. 1A, dotted line), a feature which we have not found to be backed up by any experimental data. The emergence of DP thymocytes happens only before E15.5 (Table 2 and Fig. 1B, dotted line), a finding that contradicts the experimental data^8^,^9^ which showed that fetal DP thymocytes could already be detected experimentally at stage E15. With respect to mature SP4 and SP8 thymocytes this model shows that they also emerge at a time point (just after E16.0) when they can already be detected experimentally^8^,^9^ (Table 2 and Fig. 1C,D, dotted line). There is also a delay of almost 5 days between the emergence of fetal DN and DP thymocytes, which experimental data showed to be around 3.5 days^8^,^9^, and a delay of a little more than half a day between the emergence of fetal DP thymocytes and the SP4 and SP8 thymocytes (columns SP4 and SP8 versus column DP, Table 2), which the same experimental data^8^,^9^ showed to be around one day. Thus, the model M2.V1 is unable to model the time points of emergence of the fetal DP, SP4, and SP8 thymocyte populations and the delays between DN-DP, DP-SP4, and DP-SP8, as shown in Table 2.

The change in the total number of thymocytes in the pre-natal thymus in both models is also shown (Fig. 1E). The dynamics in the composition in the four thymocyte populations of the pre-natal thymus is depicted in Fig. 1F and shows that, as the fetal thymus develops, the percentage of DN thymocytes drops while the percentage of DP ones increases, the percentage of the two populations becoming equal at around stage E17.75 (or ~1.75 days before birth), in agreement with experimental findings^9^ that showed that the fetal DP thymocyte population becomes dominant at 1–2 days before birth. By birth both the DN and DP thymocyte populations approach the normal percentage distribution that we see in the post-natal thymus.

These findings further favor the modeling approach represented by model M1.V1, an approach that models the milestones of fetal thymus development better for all four thymocyte populations.

### Modeling the development of the four main thymocyte populations in the post-natal thymus in mice

**Figure 2:**
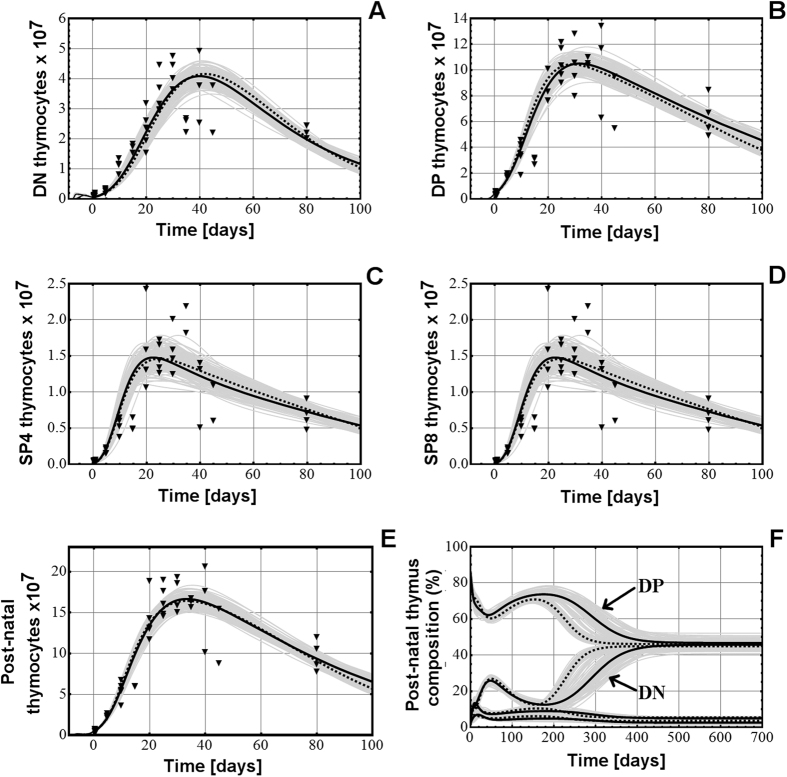
The development of the DN (**A**), DP (**B**), SP4 (**C**), and SP8 (**D**) thymocyte populations in the post-natal thymus in model M1.V1 (continuous line) and in model M2.V1 (dotted line). (**E**) The changes in the whole number of thymocytes in the post-natal thymus in both models. (**F**) The dynamics of the thymocyte composition in the post-natal thymus, also in both models. The dots on each chart are the experimental number of thymocytes at the respective time points.

We next simulated the development of the post-natal thymocyte population based on the models M1.V1 and M2.V1. The results displayed in Fig. 2A–D show the development of the four main thymocyte populations in a mouse thymus from the pre-natal emergence of each population up to 100 days after birth, using the model M1.V1 (continuous line) and the model M2.V1 (dotted line). The change in the total number of thymocytes in the post-natal thymus (Fig. 2E) is also shown. Both variants are able to model the post-natal development of all four post-natal thymocyte populations. The thymus composition of the four thymocyte populations throughout the entire post-natal life of mice (Fig. 2F) is in agreement with the experimental findings^35^, which state that the percentage of DP thymocytes drops gradually (and that of DN thymocytes increases gradually) as the mouse ages, but never drops below that of DN thymocytes. The overall dynamics of all thymocyte populations from the appearance of the first fetal DN thymocyte until the death of the animal is displayed in Supplementary Figure S2A, and is in agreement with the existing data on the gradual involution of thymus in old age^35^,^36^. The changes in each post-natal thymocyte population from 200 days up to 2 years are displayed in Figure S2B, in the Supplementary Material, and shows that, although the normal thymus supplied with a constant inflow of progenitors involutes dramatically in late age, it never ceases to operate and that thymocyte populations never reach the zero value before the age of two years, which represents the typical lifespan of a mouse.

Post-natal modeling of thymocyte population dynamics with both variants, M1.V1 and M2.V1, is able to reproduce their dynamics from birth until late age and the plots obtained by mathematical modeling match the experimental data.

### “Shock of birth” effect on the proliferation rates of all four thymocyte populations

**Figure 3:**
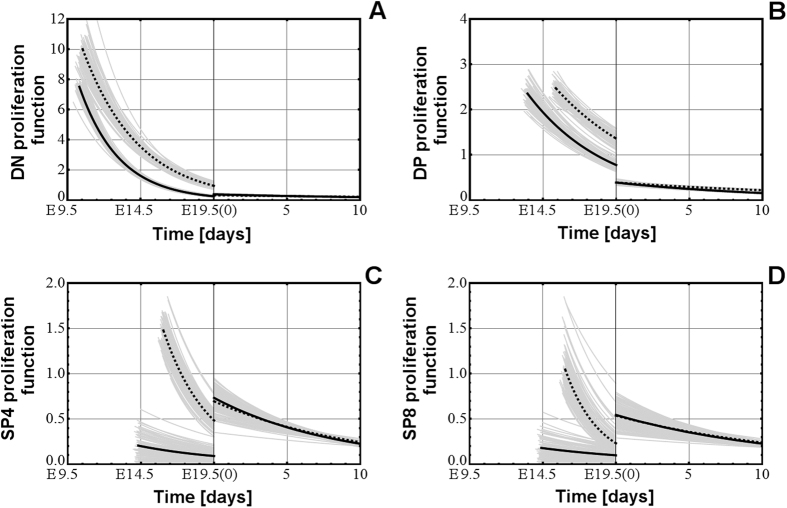
The discontinuity at birth in the proliferation rates of the DN (**A**), DP (**B**), SP4 (**C**), and SP8 (**D**) thymocyte populations in the pre-natal and post-natal period, in model M1.V1 (continuous line) and in model M2.V1 (dotted line). The vertical, thickened line at 19.5(0) represents the borderline between the end of pregnancy and the beginning of the post-natal life.

Both variants of the models - M1.V1 and M2.V1 - show that the highest proliferation rate in the pre-natal thymus occurs in the early stages of DN thymocyte formation and then their proliferation drops gradually as the thymus progresses towards birth (Fig. 3A). A similar proliferation curve but of smaller amplitude happens in case of DP thymocytes (Fig. 3B). That is consistent with the major buildup of these two thymocyte populations that happens in the pre-natal thymus. However, in case of SP4 and SP8 thymocytes, their pre-natal proliferation appears to be weaker than the post-natal one (Fig. 3C,D).

On the other hand there are experimental findings suggesting that SP4 and SP8 thymocytes retain some proliferative capacity after selection, both in pre-natal and post-natal mice^14^,^15^, and our models showed that the proliferation of both SP4 and SP8 thymocytes is significantly higher immediately after birth than in the fetal period, but only for a limited time, suggesting that own SP4 and SP8 proliferation plays an important role in the generation of immuno-competent cells in the early stages of the post-natal life, in agreement with published observations on the proliferation ability of SP4 thymocytes in neo-natal mice^14^.

One striking feature of proliferation in all thymocyte populations is their discontinuity during birth. The most intense effect is on the proliferation of DP thymocytes, which fell during birth, and on the proliferation of SP4 and SP8, which both shot up during birth. By imposing the continuity of the proliferation functions on the model M1 a reduction in the fitting quality was observed (Table 1, variant M1.V2). On the other hand, for the models M2 the discontinuity does not influence significantly the fitting quality (Table 1, variant M2.V2), but in this last model the matching to the time points of emergence of fetal thymocyte populations is poor (Table 2).

These results suggest that birth resets the development of the thymus for the post-natal period and these changes in the proliferation rates before and after birth are supported by experimental observations^14^,^15^. The models also suggest that proliferation rate plays a different role in the formation of SP4 and SP8 thymocyte populations in the pre-natal and post-natal periods.

### Analysis of the dependence of the post-natal thymus on bone marrow progenitors

We next analyzed the dependence of the post-natal thymus on the inflow of bone marrow progenitors in the first 200 days of post-natal life. Data from the literature^20^,^21^,^22^, suggest that the daily inflow of bone marrow progenitors into the post-natal thymus is constant. To analyze the impact of post-natal progenitors we examined two scenarios:

- The number of bone marrow progenitors remains constant, at the birth level for the remainder of the adult life of the mouse.
- The number of bone marrow progenitors is set to zero at birth for the remainder of the adult life of the mouse.

Both scenarios were simulated using the model characterized by decreasing proliferation rates (M1.V1) and these results are displayed below. Similar results were obtained using the model which also involves density-based control of growth (M2.V1), as both models behave similarly in post-natal life.

**Figure 4:**
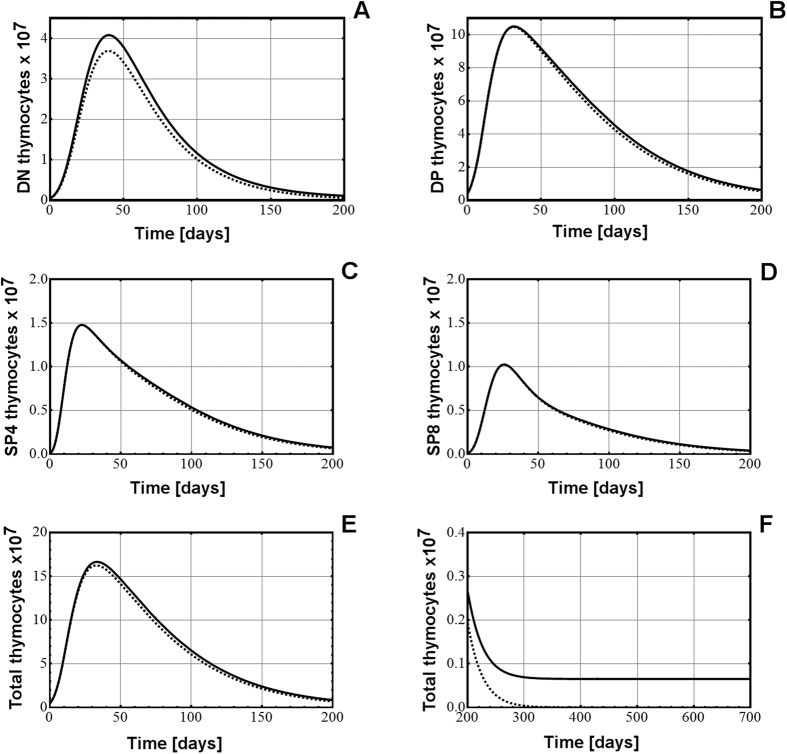
The relationship between the bone marrow progenitors and the post-natal thymus homeostasis, examined by the model M1.V1. (**A**) The impact of completely eliminating the bone marrow progenitors in the post-natal life (by setting b(t) = 0 on the DN thymocyte population). The same impact on the DP (**B**), SP4 (**C**), SP8 (**D**) thymocyte populations, and total number of thymocytes in the thymus (**E**). (**F**) the changes in the total number of thymocytes in the post-natal thymus, between 200–700 days. In each chart the continuous, thin black line is the numerical change in the thymocyte populations with a constant inflow of progenitors at the level at birth (b(t) = b_0_), and the dotted curve is the change in the thymocyte populations with post-natal progenitors set at zero (b(t) = 0).

The results displayed in Fig. 4 and Table 3 show that, in comparison with the dynamics of thymocyte populations in the thymus fueled by bone marrow progenitors (continuous line), by reducing the number of bone marrow progenitors to zero (b(t) = 0) for the remainder of the post-natal life the impact on the DN population in the first three months of life is minor (Fig. 4A, dotted line, and Table 3), and that coincides with the maximum level of activity of the thymus. The complete absence of bone marrow progenitors in the first 6 months of post-natal life has even less effect upon the DP, SP4, and SP8 populations (Fig. 4B–D, continuous lines versus dotted ones, and Table 3), suggesting that the post-natal thymus could possess an intrinsic capacity for renewal, independent of the external input of progenitors. With respect to the total number of thymocytes, the absence of post-natal progenitors has a negligible or minor effect on it in the first six months, strengthening the possibility of a self-renewal capacity of the thymus (Fig. 4E and Table 3). The percentage differences in the number of thymocytes at several time points in post-natal life, between the model with progenitors and the one without progenitors are displayed in Table 3.

It appears that the absence of bone marrow progenitors in post-natal life did not prevent the development of the post-natal thymocyte populations. In the absence of post-natal bone marrow progenitors, the thymus has a nearly identical output of mature thymocytes for the first six months of post-natal life, its most active period, suggesting that a functional thymopoiesis could occur in the absence of these progenitors.

Table 3 shows that at 6 weeks of post-natal life, when the mouse thymus normally reaches its maximal size and functionality, in the absence of progenitors, there is a difference in the DN population of only about 10% in comparison with the version with inflow of progenitors. A difference in the DN thymocyte population between the models with or without progenitors remains at subsequent stages, suggesting that DN thymocytes are somewhat affected by the absence of progenitors. In the case of DP thymocytes at 6 weeks of post-natal life, the difference between the version with progenitors and that without progenitors is <1%, suggesting that DP thymocytes could develop, in the first weeks, even in the absence of progenitors, based on the pool of DN thymocytes present at birth in the thymus. At later stages that difference increases slightly and reaches about 10% by 6 months of post-natal life (Table 3). In case of mature SP4/SP8 thymocytes the differences between the scenarios with or without progenitors at 6 weeks of post-natal life is well below 1% suggesting that mature thymocytes could also be generated in the absence of progenitors (Table 3). At 6 months the difference increases to about 10%, suggesting that the long-term impact of the absence of progenitors on thymus output of mature thymocytes is minimal. These small differences have little relevance in functional terms in the first months of post-natal life since a mouse thymus with 150 to 155 million thymocytes instead of ~160 millions as ours have, would be considered perfectly normal. The dynamics of the total number of thymocytes from 200 to 700 days in the post-natal thymus, with or without progenitors, is displayed in Fig. 4F, which shows that, as is to be expected in the case of decreasing proliferation and constant death rates, the number of thymocytes asymptotically vanishes if there is no continuous influx of progenitors.

These results suggest that the absence of progenitors in the post-natal thymus has an impact mostly on the DN population, while its effect on the other three populations is minimal, mainly in the early stages (the first six months) of post-natal thymus formation. Even with the complete absence of progenitors we could have production of functional, mature thymocytes from that thymus, comparable with that of a normal, progenitor-fuelled thymus, in the first 6 months of post-natal life. It is likely that the pool of DN and DP thymocytes present in the thymus at birth could keep the thymus going for most of its post-natal life. The absence of bone marrow progenitors seems to have more significant effects on the thymocyte populations and on the production of mature thymocytes after 6 months of post-natal life, when the organ is normally becoming less and less active, and its cellularity and functionality are only a fraction of their peak in the first weeks of post-natal life.

## Discussion

T-cell development in the thymus is a very complex phenomenon that is only partly understood. While the post-natal thymus is readily accessible to experimental intervention, the fetal thymus, particularly at the early stages of thymus organogenesis is much more difficult to manipulate experimentally. In spite of these difficulties a picture of early thymus organogenesis emerged, with the identification of the primordial progenitors in the murine fetal liver^4^,^5^,^6^ that are transported via the blood stream to the thymus anlage, where they generate the first DN thymocyte at around E10–11, which will then proliferate and differentiate^7^, resulting in the appearance of the fetal DP thymocytes and then, the first mature SP4 and SP8 thymocytes^8^,^9^. The pre-natal thymus at the end of pregnancy contains all four major thymocyte populations that are found in a normal, functional thymus from a young mouse.

Until recently, the view that thymus function depends on a constant inflow of progenitors from the bone marrow was considered a central dogma of thymopoiesis^20^,^21^,^22^. Experimental data with transplanted thymuses suggested that resident thymocytes from the transplanted thymus are gradually replaced by the ones generated from the precursors from the bone marrow of the host^23^,^24^,^25^, but recent data in mice suggest that the post-natal thymus possesses an intrinsic renewal capacity, as suggested by the identification of thymocyte populations that could for a while sustain the T-cell development when normal thymuses are transplanted into immuno-deficient mice that lack bone marrow progenitors^26^,^27^,^28^.

We intended to address the issue of thymus developmental autonomy by developing a mathematical model that could accurately describe the development of thymocyte populations from the emergence of the first embryonic thymocyte up to the death of the animal and assess the dependence of the post-natal thymus on the influx of bone marrow progenitors. Starting from a classical compartmental model of thymocyte dynamics, corresponding to the main four thymocyte populations^29^,^30^, we adapted it and turned it into two classes of models, one characterized by an exponentially decreasing proliferation rate (M1) and the other one relying on the combination of a decreasing proliferation rate and a density-based control of the growth process (M2). Both classes of models are able to describe the development of the thymocyte populations from the stage of one DN thymocyte in the fetal thymus until late age involution and death of the animal. The models incorporate our experimental data regarding the numerical changes of pre-natal and post-natal thymocytes populations, their apoptosis, and data from the literature regarding the pre-natal and post-natal proliferation and the milestones of mouse fetal thymus development. From several sets of parameters estimated on the basis of the experimental data, we selected the ones which were in agreement with the information available in the literature. The best fit with the experimental data was obtained when different sets of parameters were used in the modeling of the pre-natal and post-natal thymocyte dynamics, suggesting that the processes corresponding to these stages are characterized by different rates. This discontinuity during birth in thymocyte dynamics is supported by experimental data, which showed that in the case of proliferation, the fetal thymus has greater proliferation in thymocytes than the post-natal thymus^12^, and the neo-natal thymus has a greater proliferation of thymocytes than the adult thymus^11^. This particular pattern of proliferation is what the models also revealed and suggests that discontinuity in proliferation rates during birth happens normally in the thymus and birth actually resets the development pattern of the thymus for the remainder of the post-natal life.

Nonetheless, there are some major differences in the ability of the models analyzed to fit the experimental data and to match specific time points in the development of thymocyte populations, primarily in the pre-natal period. In the model which involves density based control of growth (M2.V1), the onset of DP, SP4, and SP8 thymocyte development is several days later than the time point when they are already detected experimentally. Furthermore, the time delay in the transfer between fetal DN and DP compartments is much higher than the values reported in the literature^18^, while the delay between the emergence of fetal DP thymocytes and fetal SP4/SP8 mature thymocytes is much shorter than that found in experimental observations. Thus, density-based models (like M2.V1 and M2.V2) may have a comparable fit with the experimental data but they are unable to match the essential time points in the development of the fetal thymocyte populations. On the other hand the models characterized by an exponentially decreasing proliferation rate (like M1.V1) fit the experimental data and match the crucial milestones during fetal development of thymocyte populations.

When the bone marrow progenitors are absent from the development of the post-natal thymus, the thymocyte populations still form, although with some differences. The most sensitive population is the DN subset while the DP, SP4 and SP8 are far less influenced by the absence of progenitors. The total number of thymocytes in the thymus developed in the absence of bone marrow progenitors is not significantly different from the thymus developed with progenitors for the first 6 months of the post-natal life, precisely the period in which the thymus is the most active. The impact of the absence of bone marrow progenitors on the thymocyte populations becomes more significant only in the later stages of post-natal life, at a time when the thymus has lost most of its cells and its functions have diminished anyway. It is likely that DN and DP thymocyte populations resident in the thymus at birth could sustain the thymic homeostasis for many months into the post-natal life.

Although the two classes of models use different approaches to modeling the growth processes, there are several aspects which both of them reveal:

- A rapid growth of the post-natal thymus in the first weeks after birth and the subsequent age-induced involution of the organ.
- Similar distribution of thymocyte populations in the thymus both in pre-natal and post-natal periods (Figs 1F and 2F).
- The absence of bone marrow progenitors has little impact on post-natal thymus development in the first 6 months, suggesting that the thymus could contain self-renewal capability.

Based on models characterized by decreasing proliferation rates, it is possible to show that the post-natal thymus could develop quasi-normally in the first several months of life with no inflow of bone marrow progenitors, a result in agreement with some recent experimental findings that question the dependency of the post-natal thymus of a constant inflow of progenitors from the bone marrow^26^,^27^,^28^. Such mathematical models are capable of describing subtle features and inter-relations of the thymocyte dynamics in the thymus over the entire life span of the animal.

## Methods

### Animals

We used NMRI mice throughout our experiments, both female and male. The pregnancy in our experimental mice lasted 19.5 days, with the plug day considered the day 0 of pregnancy (E0.0). At indicated time points the animals were sacrificed by asphyxiation with carbon dioxide and their thymuses dissected out for further processing, as described below. The total number of thymocytes in each thymus was counted via trypan blue and a hemocytometer. The total number for each thymocyte subpopulation was obtained by multiplying the total number of thymocytes with the percentage of the respective population, as inferred from the flow cytometry dot plots. All the experiments on animals comply with European Convention for the Protection of Vertebrate Animals used for Experimental and Other Scientific Purposes (Strasbourg, France, 1986), with all its subsequent revisions, and Directive 2010/63/EU on the protection of animals used for scientific purposes. The experimental protocol was reviewed and approved by the Ethics Committee of the “Victor Babes” University of Medicine and Pharmacy Timisoara.

### Thymus dissection and flow cytometry on thymocyte suspensions

Time-mated pregnant mice were sacrificed at E17.5, E18.5, and E19.5, in the morning immediately after overnight birth (considered the time point 0 of the post-natal life). We used multiple litters of pups to sacrifice every 5 days up to day 50 after birth, and on day 80 after birth. Their thymi were dissected out and washed with cold PBS to remove the traces of blood. Then with the help of a syringe piston it was minced through a sterile wire mesh immersed in cold PBS. The thymocyte suspension underneath was recovered, centrifuged at 700 g and washed twice with cold PBS. Concentration of cells was adjusted to 1 × 10^6^ cells/ml. The cells were labeled with FITC- and APC-conjugated, rat anti-mouse CD4, CD8 antibodies (Southern Biotech, Birmingham, USA) and propidium iodide for 30 minutes in the dark, at 4 °C and then washed twice with cold PBS. The cells were re-suspended in PBS and analyzed on a BD FACSCalibur cytometer with at least 20,000 events recorded. Apoptotic thymocytes were identified as propidium iodide-positive cells with lower forward scattering while the four thymocyte populations were identified based on CD4 and CD8 antibody staining. The data was analyzed and displayed with the WinMDI and FCS Express software packages (De Novo Software, Los Angeles, USA).

### Mathematical modeling

**Figure 5:**
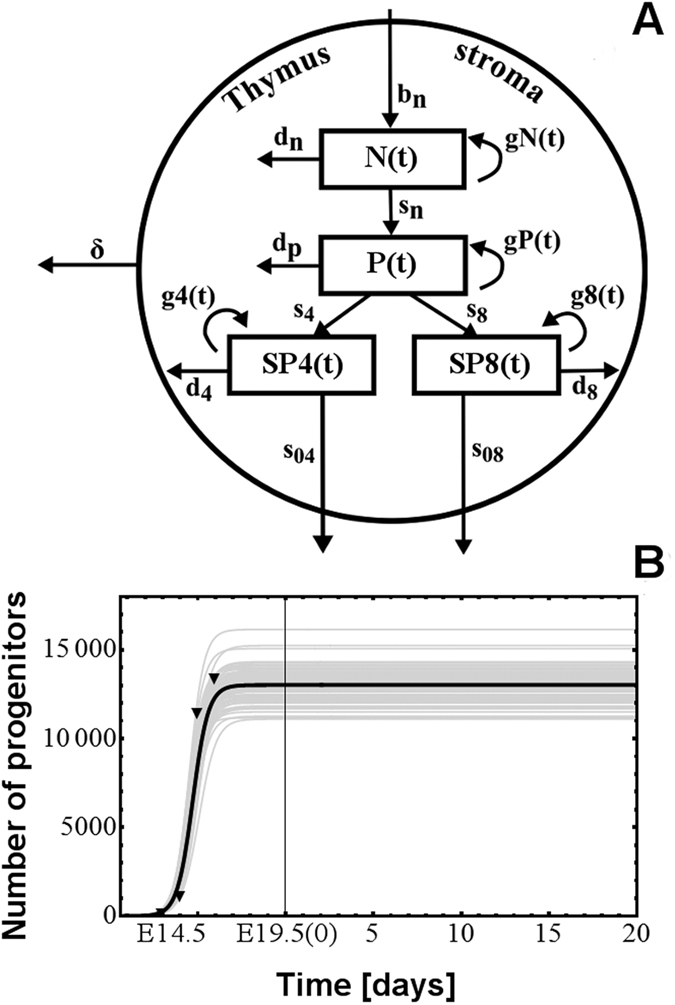
(**A**) The conveyor-belt type, compartmental model of the pre-natal and post-natal thymus, used in our simulations. The stromal component of the thymus surrounds the thymocytes, as it happens *in vivo*. (**B**) logistic functions fitted to the number of thymic progenitors in the pre-natal thymus^6^. The vertical, thickened line at 19.5(0) represents the borderline between the end of pregnancy and the beginning of the post-natal life.

Mathematical models involving density-based regulation of the growth process^29^ are appropriate in modeling the homeostasis, but as long as they rely on constant proliferation rates they cannot model the involution. Aiming to describe the natural involution in the size of thymocyte populations we proposed to use time decreasing proliferation rates. The compartmental model we used contained a compartment for each of the four main thymocyte populations (DN, DP, SP4, and SP8), and we added an extra-compartment that drained all the dead thymocytes from the other four compartments (Fig. 5A). The dynamics of these thymocyte populations - (N(t), P(t), SP4(t), SP8(t) and apoptotic cells - D(t)) - in both the fetal and post-natal time periods were modeled by a system of five differential equations, as described in Eq. (1).


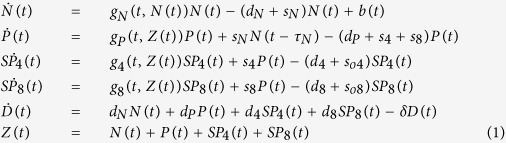


The first four equations describe the rate of change in the number of thymocytes, based on typical terms describing proliferation, death and transfer, while the fifth equation models the compartment of apoptotic cells. This last compartment receives as input apoptotic cells from all the other compartments, as it is reflected by the first four terms of the fifth differential equation. The process of degradation of the apoptotic cells is modeled by the last term, where *δ* is the degradation rate. The compartment corresponding to apoptotic cells has been introduced in order to decouple the transfer and death rates that appear in the other compartments and to reduce the degree of parameter non-identifiability. It is primarily a mathematical compartment, since dead thymocytes are not a typical population undergoing some sort of biological development. Each of the first four equations in Eq. (1) contains a term which models the proliferation process. We considered two main classes of models: one characterized by exponentially decreasing proliferation rates, as described in Eq. (2), hereafter identified as M1, and another one in which the growth term involves a density related factor as well as a decreasing proliferation rate, as described in Eq. (3), noted as M2.


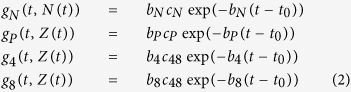



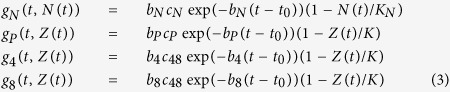


In Eqs (2) and (3) the parameter *t*_*0*_ represents the time moment the first thymic progenitor activates the process of fetal thymus growth, around stage E10.5. The parameters *b*_*N*_, *b*_*P*_, *b*_*4*_ and *b*_*8*_ control the deceleration of the proliferation rates while the values of the parameters *c*_*N*_, *c*_*P*_ and *c*_*48*_ are related to the ratio between the number of thymocytes corresponding to the last stage of development and that corresponding to the first stage. Parameters *K*_*N*_ and *K* in Eq. (3) correspond to carrying capacities and are specific to density based control of population growth^29^. The last term, *b(t)* in the equation describing the dynamics of *N(t)* corresponds to the influx of thymic progenitors. By combining Eq. (1) with Eq. (2) a simple model was obtained which has as its main characteristic the exponential decrease of the proliferation rate. This would correspond to the case when the involution is modeled only as an effect of ageing. When Eq. (1) are combined with Eq. (3) we arrive to a model describing the growth and involution processes as an interplay between density-based control and age-induced deceleration of the proliferation factor.

By fitting the data on thymocyte precursors during the fetal period^19^ we found that a logistic function accurately captures the variation in data (Fig. 5B). Consequently, in simulations we considered that the number of thymic progenitors varies according to the function given in Eq. (4). The parameters of function *b(t)* are estimated from data together with the other parameters of the model but such that *b*(*t*_*0*_) = *10*^−7^ (this corresponds to the assumption that *t*_*0*_ denotes the time at which the first thymic progenitors activate the process of the thymus formation).


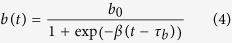


As the differentiation process of DN thymocytes in DP thymocytes requires several days^37^ we included in the model a delay (denoted by *τ*_*N*_ in Eq. (1)), corresponding to the transfer of cells from the DN to the DP compartment. In order to keep the model simple we ignored the significantly smaller delay arising in the process of differentiating SP4 and SP8 thymocytes from DP ones.

These two classes of models involve several groups of parameters which have to be estimated in order to construct a model reflecting the behavior illustrated by the experimental data:

- Parameters involved in the proliferation process: *b*_*N*_. *b*_*P*_, *b*_*4*_, *b*_8_, *c*_*N*_, *c*_*P*_ and *c*_48_;
- Transfer and export rates: *s*_*N*_, *s*_4_, *s*_8_, *s*_04_ and *s*_08_;
- Death rates: *d*_*N*_, *d*_*P*_, *d*_4_ and *d*_8_;
- Degradation rate for the apoptotic cells: *δ*;
- Time delay corresponding to the differentiation of double positive cells from double negative cells: *τ*_*N*_;
- Time at which (during the fetal period) the first thymic progenitor initiates the thymopoiesis: *t*_0_;
- Carrying capacities, *K*_*N*_ and *K*, used in the density-based control of populations growth;
- Parameters controlling the variation of the number of thymic progenitors: *β*, *b*_0_, and *τ*_*b*_. It should be noted that the parameter *β* arising in Eq. (4) is computed on the basis of the estimated values of *b*_0_ and *τ*_*b*_ such that the number of progenitors at *t*_0_ is 1.

As our aim was to model the overall dynamics of thymocyte populations from the fetal period until death of the animal, the first issue in modeling was to analyze whether the same parameter values are appropriate for both the pre-natal and post-natal periods. In order to answer this question we conducted some preliminary analysis for each of the two classes of models, considering both the case when the same set of parameters is used in pre- and post-natal stages and the case when there are no constraints imposed on the parameters values, i.e. the parameters values may have pre-natal and post-natal differences. The main point to notice is that by applying the same parameters in pre- and post-natal stages the quality of the models is significantly reduced, as illustrated by the mean squared error (MSE) values presented in Table 4.

Table 4 shows that for both classes of models the best fitting is obtained when different sets of parameters are used in the pre-natal and post-natal periods. In such a case a discontinuity may appear at the moment of birth in the function describing the decreasing proliferation rate. Therefore we further analyzed whether imposing a constraint concerning the continuity of the proliferation function improves or diminishes the quality of the model. The imposed constraint refers to the post-natal values of *c*_*N*_, *c*_*P*_, *c*_48_, which are computed directly using the corresponding pre-natal values, i.e. *c*_*postnatal*_ = *b*_*prenatal*_ *c*_*prenatal*_ exp(*b*_*prenatal*_*t*_0_), the numbers of parameters to be estimated being, in this way, decreased by three. Thus we arrived at four variants of the mathematical modeling, corresponding to the combination between two classes of models, which either incorporate (M1) or do not incorporate (M2) density-based control of the population growth, each with two variants, which allow (V1) or do not allow a discontinuity (V2) in the proliferation function at the moment of birth. In the Results section, out of the two main classes of models we used only the variants characterized by different sets of parameters in pre-natal and post-natal stages (M1.V1 and M2.V1, respectively). The models were separated initially on the basis of their fitting quality, and the models with poorer fitting were not pursued further. Models with comparable fitting were examined for their ability to model the dynamics of the thymocyte populations and to match crucial developmental time points in the development of the fetal thymus, from our data or from the literature.

The calibration of the models to experimental data was done by minimizing the mean squared error using a stochastic optimization procedure based on an evolutionary algorithm hybridized with a Nelder-Mead local optimization method^38^. In order to collect statistical values and construct confidence intervals, the parameters were re-estimated by using a bootstrapping approach (re-sampling the experimental data). All results presented in this paper are based on 100 re-estimation steps. Search ranges and estimated confidence intervals are presented in Table SI and Figure S1 in the Supplementary Material. It should be noted that in the estimation process of the parameters corresponding to the post-natal stage we imposed a constraint on the transfer and death rates corresponding to DP subset (*s*_*N*_ > *d*_*P*_ + *s*_4_ + *s*_8_), ensuring that the number of DP thymocytes did not become smaller than that of DN thymocytes in the late stages of post-natal life, in agreement with the experimental findings^37^.

The gray curves on the charts (Figs 1, 2, 3, 4 and 5 and Supplementary Figures) correspond to parameter values obtained in the 100 stages of bootstrapping based re-estimation starting from a set of parameters selected out of 10 preliminary runs of the stochastic parameter estimation. Similarly, the black curve in each chart corresponds to the average values of the parameters calculated from the set of 100 values obtained by bootstrapping. These average values were used in the subsequent analysis and scenarios of thymocyte dynamics. On each applicable chart the black triangles represent the experimental data. The models with these average values of the parameters have been validated with respect to data reported in the literature in relation to the following aspects: shape of fetal and postnatal dynamics of thymocyte populations; thymus composition; proliferation in the fetal versus postnatal thymus; fetal and postnatal delays in the transfer from the DN compartment to the DP one; onset of embryonic thymopoiesis; and logistic shape of the number of precursors in the fetal thymus (details in Tables SI and SII from Supplementary Material). The parameter estimation procedure was fully implemented in Mathematica 7.0 (Wolfram Research, Oxfordshire, UK), which was also used for all simulations and graphs.

## Additional Information

**How to cite this article**: Zaharie, D. *et al*. Modeling the development of the post-natal mouse thymus in the absence of bone marrow progenitors. *Sci. Rep.* **6**, 36159; doi: 10.1038/srep36159 (2016).

**Publisher’s note:** Springer Nature remains neutral with regard to jurisdictional claims in published maps and institutional affiliations.

## Supplementary Material

Supplementary Information

## Figures and Tables

**Table 1:** Fitting quality of the four model variants (MSE- Mean Squared Error, AIC – Akaike Information Criterion).

Measure	Model M1		Model M2	
	V 1	V 2	V 1	V 2
MSE	0.0149 ± 0.0020	0.0189 ± 0.0029	0.0154 ± 0.0020	0.0150 ± 0.0022
AIC	−1180.40 ± 55.28	−1087.93 ± 59.16	−1156.94 ± 55.74	−1166.96 ± 58.65
Number of estimated parameters	39	36	43	40

Smaller values of MSE and AIC correspond to higher quality models.

**Table 2:** Confidence intervals for the time moment (days of gestation) of the first cell appearance, for each thymocyte population, in the pre-natal thymus.

Model	DN, embryo stage	DP, embryo stage	SP4, embryo stage	SP8, embryo stage
M1.V1	E10.29–10.35	E13.43–13.49	E14.30–14.35	E14.35–14.41
M1.V2	E10.65–10.75	E13.46–13.55	E14.27–14.35	E14.23–14.32
M2.V1	E10.50–10.58	E15.34–15.41	E16.02–16.07	E16.00–16.06
M2.V2	E10.75–10.79	E15.93–15.93	E16.48–16.52	E16.49–16.53

**Table 3:** Percentage differences ((population size with progenitors − population size without progenitors)/(population size with progenitors)*100%) between the thymocytes populations developed in the presence or absence of bone marrow progenitors.

Thymocytes population	6 weeks (%)	3 months (%)	6 months (%)	1 year (%)	18 months (%)	2 years (%)
DN	9.62	11.78	34.97	98.47	99.98	99.99
DP	0.96	5.04	11.23	80.92	99.57	99.99
SP4	0.55	4.56	10.54	78.51	99.50	99.99
SP8	0.39	4.21	10.19	76.92	99.41	99.99
Total thymocytes	3.09	6.28	14.10	87.54	99.75	99.99

**Table 4:** Comparison between the fitting quality of the two main classes of models (M1 and M2) in the case when the parameter values are allowed to differ in the pre-natal and post-natal stages (V1) or the same set of parameters is used in both stages (V2).

Models/Variants	V1	V2
M1	MSE: 0.0149 ± 0.0020	MSE: 0.0617 ± 0.0066
M2	MSE: 0.0154 ± 0.0020	MSE: 0.0833 ± 0.0075

The reported values are averages and standard deviations of MSE (mean squared error) computed by fitting the models to several experimental data samples. The lower the value of MSE the better the fitting.

## References

[b1] Pearse G. Normal Structure, Function and Histology of the Thymus. Toxicol. Pathol. 34, 504–514 (2006). 17067941 10.1080/01926230600865549

[b2] Petrie H. T., Pearse M., Scollay R. & Shortman K. Development of immature thymocytes: initiation of CD3, CD4, and CD8 acquisition parallels down-regulation of the interleukin 2 receptor alpha chain. Eur J Immunol. 20, 2813–2815 (1990). 2148525 10.1002/eji.1830201243

[b3] Gordon J. & Manley N. R. Mechanisms of thymus organogenesis and morphogenesis. Development. 138, 3865–3878 (2011). 21862553 10.1242/dev.059998 PMC3160085

[b4] Itoi M., Kawamoto H., Katsura Y. & Amagai T. Two distinct steps of immigration of hematopoietic progenitors into the early thymus anlage. Int. Immunol. 13, 1203–1211 (2001). 11526101 10.1093/intimm/13.9.1203

[b5] Owen J. J. & Ritter M. A. Tissue interaction in the development of thymus lymphocytes. J. Exp. Med. 129, 431–442 (1969). 5762051 10.1084/jem.129.2.431 PMC2138610

[b6] Dunon D., Allioli N., Vainio O., Ody C. & Imhof B. A. Quantification of T-cell progenitors during ontogeny: thymus colonization depends on blood delivery of progenitors. Blood. 93, 2234–2243 (1999). 10090932

[b7] Ikawa T. . Identification of the earliest prethymic T-cell progenitors in murine fetal blood. Blood. 103, 530–537 (2004). 14512296 10.1182/blood-2003-06-1797

[b8] Fairchild P. J. & Austyn J. M. Developmental changes predispose the fetal thymus to positive selection of CD4+CD8− T cells. Immunology. 85, 292–298 (1995). 7642219 PMC1383894

[b9] Xiao S. Y., Li Y. & Chen W. F. Kinetics of thymocyte developmental process in fetal and neonatal mice. Cell Research 13, 265-27 (2003). 10.1038/sj.cr.7290171 12974616

[b10] Gray D. H. . Developmental kinetics, turnover, and stimulatory capacity of thymic epithelial cells. Blood 108, 3777–3785 (2006). 16896157 10.1182/blood-2006-02-004531

[b11] Cuddihy A. R. . VEGF-mediated cross-talk within the neonatal murine thymus. Blood 113, 2723–2731 (2009). 19088378 10.1182/blood-2008-06-162040 PMC2661859

[b12] Su D. M. & Manley N. R. Stage-specific changes in fetal thymocyte proliferation during the CD4−8− to CD4+8+ transition in wild type, Rag1−/−, and Hoxa3, Pax1 mutant mice. BMC Immunol. 19, 12–22 (2002). 10.1186/1471-2172-3-12 PMC130029 12241558

[b13] Egerton M., Scollay R. & Shortman K. Kinetics of mature T-cell development in the thymus. Proc. Natl. Acad. Sci. USA 87, 2579–2582 (1990). 2138780 10.1073/pnas.87.7.2579 PMC53733

[b14] Ceredig R. & Waltzinger C. Neonatal mouse CD4+ mature thymocytes show responsiveness to interleukin 2 and interleukin 7: growth *in vitro* of negatively selected V beta 6- and V beta 11-expressing CD4+ cells from (C57BL/6 x DBA/2)F1 mice. Int. Immunol. 2, 869–877 (1990). 2149068 10.1093/intimm/2.9.869

[b15] Chow K. P. . Selective reduction of post-selection CD8 thymocyte proliferation in IL-15Rα deficient mice. PLoS One 7, e33152 (2012). 22448237 10.1371/journal.pone.0033152 PMC3308975

[b16] Rothenberg E. V. Death and transfiguration of cortical thymocytes: a reconsideration. Immunol. Today 11, 116–119 (1990). 2187467 10.1016/0167-5699(90)90047-d

[b17] Matsumoto K. . Two differential pathways from double-negative to double-positive thymocytes. Immunology 72, 20–26 (1991). 1825481 PMC1384330

[b18] Rodewald H. R. . Fc gamma RII/III and CD2 expression mark distinct subpopulations of immature CD4−CD8− murine thymocytes: *in vivo* developmental kinetics and T cell receptor beta chain rearrangement status. JEM. 177, 1079–1092 (1993). 10.1084/jem.177.4.1079 PMC2190966 8096236

[b19] Douagi I., André I., Ferraz J. C. & Cumano A. Characterization of T cell precursor activity in the murine fetal thymus: evidence for an input of T cell precursors between days 12 and 14 of gestation. Eur. J. Immunol. 30, 2201–2210 (2000). 10940911 10.1002/1521-4141(2000)30:8<2201::AID-IMMU2201>3.0.CO;2-2

[b20] Mori S., Shortman K. & Wu L. Characterization of thymus-seeding precursor cells from mouse bone marrow. Blood 98, 696–704 (2001). 11468169 10.1182/blood.v98.3.696

[b21] Perry S. S. . L-selectin defines a bone marrow analog to the thymic early T-lineage progenitor. Blood 103, 2990–2996 (2004). 15070675 10.1182/blood-2003-09-3030

[b22] Scott Perry S., Pierce L. J., Slayton W. B. & Gerald J. S. Characterization of Thymic Progenitors in Adult Mouse Bone Marrow. Journal of Immunology 170, 1877–1886 (2003). 10.4049/jimmunol.170.4.1877 12574354

[b23] Berzins S. P., Boyd R. L. & Miller J. F. The role of the thymus and recent thymic migrants in the maintenance of the adult peripheral lymphocyte pool. J. Exp. Med. 187, 1839–1848 (1998). 9607924 10.1084/jem.187.11.1839 PMC2212318

[b24] Frey J. R., Ernst B., Surh C. D. & Sprent J. Thymus-grafted SCID mice show transient thymopoiesis and limited depletion of V beta 11+ T cells. J. Exp. Med. 175, 1067–1071 (1992). 1532413 10.1084/jem.175.4.1067 PMC2119181

[b25] Takeda S., Rodewald H. R., Arakawa H., Bluethmann H. & Shimizu T. MHC class II molecules are not required for survival of newly generated CD4+ T cells, but affect their long-term life span. Immunity 5, 217–228 (1996). 8808677 10.1016/s1074-7613(00)80317-9

[b26] Peaudecerf. L. . Thymocytes may persist and differentiate without any input from bone marrow progenitors. J Exp Med. 209, 1401–1408 (2012). 22778388 10.1084/jem.20120845 PMC3420331

[b27] Martins V. C. . Thymus-autonomous T cell development in the absence of progenitor import. J. Exp. Med. 209, 1409–1417 (2012). 10.1084/jem.20120846 PMC3420332 22778389

[b28] Boehm T. Self-renewal of thymocytes in the absence of competitive precursor replenishment. J. Exp. Med. 209, 1397–1400 (2012). 22851642 10.1084/jem.20121412 PMC3420333

[b29] Mehr R., Globerson A. & Perelson A. S. Modeling positive and negative selection and differentiation processes in the thymus. J. Theor. Biol. 175, 103–126 (1995). 7564390 10.1006/jtbi.1995.0124

[b30] Mehr R., Perelson A. S., Fridkis-Hareli M. & Globerson A. Feedback regulation of T cell development in the thymus. J. Theor. Biol. 181, 157–167 (1996). 8935593 10.1006/jtbi.1996.0122

[b31] Thomas-Vaslin V., Altes H. K., de Boer R. J. & Klatzmann D. Comprehensive assessment and mathematical modeling of T cell population dynamics and homeostasis. J. Immunol. 180, 2240–2250 (2008). 18250431 10.4049/jimmunol.180.4.2240

[b32] Morpurgo D., Serenthà R., Seiden P. E. & Celada F. Modeling thymic functions in a cellular automaton. Int. Immunol. 7, 505–516 (1995). 7547676 10.1093/intimm/7.4.505

[b33] Sawicka M. . From pre-DP, post-DP, SP4, and SP8 Thymocyte Cell Counts to a Dynamical Model of Cortical and Medullary Selection. Front. Immunol. 14, 1–14 (2014). 10.3389/fimmu.2014.00019 PMC3924582 24592261

[b34] Zhang Y., Finegold M. J., Jin Y. & Wu M. X. Accelerated transition from the double-positive to single-positive thymocytes in G alpha i2-deficient mice. Int. Immunol. 17, 233–243 (2005). 15684040 10.1093/intimm/dxh204

[b35] Hale J. S., Boursalian T. E., Turk G. L. & Fink P. J. Thymic output in aged mice. Proc. Natl. Acad. Sci. USA 103, 8447–8452 (2006). 16717190 10.1073/pnas.0601040103 PMC1482512

[b36] Vallejo A. N. . Resistance to age-dependent thymic atrophy in long-lived mice that are deficient in pregnancy-associated plasma protein A. Proc Natl Acad Sci. USA 106, 11252–11257 (2009). 19549878 10.1073/pnas.0807025106 PMC2700140

[b37] Shortman K., Egerton M., Spangrude G. J. & Scollay R. The generation and fate of thymocytes. Semin. Immunol. 2, 3–12 (1990). 2129900

[b38] Moleriu R. D. . Insights into the mechanisms of thymus involution and regeneration by modeling the glucocorticoid-induced perturbation of thymocyte populations dynamics. J. Theor. Biol. 348, 80–99 (2014). 24486233 10.1016/j.jtbi.2014.01.020

