# Supplementary material for: Modeling the development of the post-natal mouse thymus in the absence of bone marrow progenitors
Source: Sci Rep. 2016 Nov 8;6:36159. doi: 10.1038/srep36159 (PMC5099910; doi:10.1038/srep36159)
Supplement: Supplementary Information [file srep36159-s1.doc]

**Modeling the development of the post-natal mouse thymus in the absence of bone marrow progenitors.**

Daniela Zaharie ^‡^, Radu D. Moleriu, ^‡ ,*^, Felix A. Mic^†^

^‡^Faculty of Mathematics and Computer Science, West University of Timisoara, 4 Vasile Parvan Blvd., ^†^Department of Functional Sciences, “Victor Babes” University of Medicine and Pharmacy, 2 Eftimie Murgu Sq. Timisoara, Romania.

Supplemental Table SI.

| *Parameter* | *Notation* | *Search range (fetal)* | *95% confidence interval*  *(fetal)* | *Search range (post-natal)* | *95% confidence interval*  *(post-natal)* |
| --- | --- | --- | --- | --- | --- |
| Proliferation parameters^29,30^ |  | (0,0.5) | (0.36,0.37)  (0.35,0.36)  (0.26,0.27)  (0.418,0.422) | (0,0.5) | (0.06,0.07)  (0.032,0.033)  (0.036,0.037)  (0.037,0.038) |
|  |  | (0,0.5) | (0.18,0.19)  (0.37,0.38)  (0.14,0.15)  (0.339,0.343) | (0,0.5) | (0.08,0.09)  (0.10,0.11)  (0.056,0.058)  (0.056,0.057) |
|  |  | (0,0.5) | (0.15,0.16)  (0.30,0.31)  (0.32,0.33)  (0.333,0.339) | (0,0.5) | (0.11,0.12)  (0.033,0.036)  (0.102,0.108)  (0.086,0.090) |
|  |  | (0,0.5) | (0.11,0.12)  (0.27,0.28)  (0.43,0.44)  (0.333,0.339) | (0,0.5) | (0.08,0.09)  (0.039,0.042)  (0.078,0.084)  (0.086.0.090) |
|  |  | (0,50) | (22.71,22.83)  (21.63,21.80)  (40.41,41.05)  (36.24,36.59) | (0,10) | (5.88,5.98)  (9.43,9.55)  (8.77,8.91)  (9.42,9.56) |
|  |  | (0,50) | (23.70,24.39)  (42.72,43.88)  (35.33,36.00)  (23.26,23.81) | (0,10) | (4.26,4.34)  (5.04,5.15)  (6.78,6.89)  (6.64.6.75) |
|  |  | (0,50) | (2.25,2.88)  (14.85,15.80)  (28.82,29.67)  (36.10,36.76) | (0,10) | (6.18,6.29)  (8.29,8.52)  (6.57,6.70)  (6.83,6.96) |
| Transfer rates^29,30^ |  | (0,0.5) | (0.38,0.39)  (0.41,0.42)  (0.38,0.39)  (0.45,0.46) | (0,0.5) | (0.027,0.029)  (0.065,0.067)  (0.044,0.045)  (0.044,0.045) |
|  |  | (0,0.1) | (0.04,0.05)  (0.035,0.037)  (0.037,0.039)  (0.024,0.025) | (0,0.1) | (0.017,0.018)  (0.011,0.013)  (0.017,0.018)  (0.024,0.025) |
|  |  | (0,0.1) | (0.028,0.03)  (0.045,0.047)  (0.041,0.042)  (0.021,0.022) | (0,0.1) | (0.006,0.007)  (0.019,0.021)  (0.021,0.022)  (0.014,0.015) |
| Export rates^29,30^ |  | (0,1) | (0.73,0.75)  (0.71,0.72)  (0.66,0.68)  (0.39,0.41) | (0,1) | (0.003,0.006)  (0.097,0.114)  (0.009,0.015)  (0.004,0.007) |
|  |  | (0,1) | (0.31,0.33)  (0.46,0.48)  (0.65,0.66)  (0.16,0.18) | (0,1) | (0.05,0.07)  (0.13,0.15)  (0.082,0.099)  (0.08, 0.10) |
| Death rates^29,30^ |  | (0,1) | (0.42,0.43)  (0.23,0.25)  (0.67,0.68)  (0.59,0.60) | (0,1) | (0.0003,0.001)  (0.0009,0.0020)  (0.0003,0.0007)  (0.0007,0.001) |
|  |  | (0,1) | (0.16,0.17)  (0.08,0.10)  (0.27,0.28)  (0.29,0.30) | (0,1) | (0.0021,0.0034)  (0.013,0.016)  (0.0037,0.0049)  (0.0043,0.0057) |
|  |  | (0,1) | (0.83,0.85)  (0.19,0.22)  (0.80,0.82)  (0.55,0.56) | (0,1) | (0.16,0.17)  (0.11,0.13)  (0.13,0.14)  (0.19,0.20) |
|  |  | (0,1) | (0.11,0.14)  (0.63,0.65)  (0.42,0.44)  (0.39,0.41) | (0,1) | (0.06,0.07)  (0.08,0.10)  (0.18,0.20)  (0.10,0.11) |
| Degradation rate of apoptotic cells^29,30^ | $\delta$ | (0,6) | (0.94,1.01)  (0.29,0.37)  (1.63,1.71)  (1.71,1.79) | (0,6) | (0.15,0.16)  (0.19,0.21)  (0.20,0.21)  (0.21,0.22) |
| Delay in the transfer between DN and DP [days]^29,31^ |  | (2,5) | (2.77,2.80)  (2.48,2.53)  (4.49,4.53)  (4.92,4.95) | (10,14) | (12.96,13.34)  (11.84,11.93)  (12.30,12.36)  (12.91,12.96) |
| Fetal stage when the thymopoiesis is initiated^8^ |  | (E7.5 - 13.5) | (9.99 - 10.05)  (10.36 - 10.46)  (10.25 - 10.33)  (10.53 - 10.57) | N/A | N/A |
| Maximal number of progenitors [10^7^ cells]^29,30^ |  | (10^-5^, 2∙10^-3^) | (0.0012859, 0.00131817)  (0.0002457, 0.0002917)  (0.00172126, 0.0018004)  (0.00133144, 0.00135302) | N/A | N/A |
| Inflection point of the logistic function describing the dynamics of the fetal progenitors^7,19^ |  | (E13.5 - 16.5) | (14.19-14.24)  (15.06-15.13)  (15.16-15.20)  (15.53-15.56) | N/A | N/A |
| Slope of the logistic function describing the dynamics of the fetal progenitors^7,19^ | $\beta$ | N/A  (it is computed  such that  *b(t0)*=10^-7^ | (2.24, 2.28)  (1.63, 1.70)  (1.98, 2.02)  (1.89, 1.91) | N/A | N/A |
| Carrying capacities for the models with density based control of the growth^29,30^ | $K$ | (0,1)  (0,2) | (0.20, 0.22)  (0.88, 0.91) | (5, 15)  (25, 40) | (11.7, 11.9)  (32.4, 32.6) |

Supplemental Table SI. Parameters of the mathematical models: search ranges and estimated confidence intervals (by bootstrapping, using a sample size of 100) for pre-natal and post-natal periods. N/A denotes cases when the parameter is identical for pre-natal and post-natal periods. The reported values correspond to the four variants of the model (i) M1.V1; (ii) M1.V2; (iii) M2.V1 (iv) M2.V2, in this order from top to bottom, in each row of the columns displaying the 95% confidence interval.

**Supplemental Table SII.**

| ***Characteristics of the***  ***mathematical model*** | ***Findings from the simulations of our model*** | ***Supporting results reported in the literature*** |
| --- | --- | --- |
| Decreasing proliferation modeled through exponentially decreasing rates, both in the fetal and the post-natal stages | Fitted dynamics of thymocyte populations is in accordance with the experimental data corresponding to fetal and post-natal development (Figures 1, 2) | Same overall dynamics of fetal and post-natal thymocyte populations is reported in Ref. (8), Figure 1A. Decreased proliferation from neonatal to adult thymus has also been reported in Ref. (24), Figure 1A |
|  | The DP population becomes dominant at E17.75 (or 1.75 days before birth) | According to the experimental data reported in Ref. (9), Table 1, the inversion between the fetal subsets of DN and DP takes place between 1-2 days before birth |
|  | The percentage of the DN population at E15.5 is around 98% of the total thymocytes | According to Refs. (9, 17) DN thymocytes represent at least 95% of the total thymocytes at E15.5 |
| Different parameters in the fetal and the post-natal stages | Higher proliferation of immature thymocytes in the fetal versus the adult thymus and discontinuity at birth in the function describing the proliferation factor (Figure 3) | Ref. (3), Figure 1F states that fetal proliferation of DN and DP population is higher than in the adult thymus |
| Logistic dynamics of the number of progenitors in the fetal thymus | The number of progenitors significantly increases until 3 days before birth, then it remains almost constant (Figure 5B) | Our estimated logistic function approximates well to the experimental data reported in Ref. (19), Table 1, Figure 2B |
| Delay in the transfer between DN and DP compartments (different in the fetal and the post-natal stages) | The estimated delay in the post-natal stage is around 13.25 days while for the fetal stage it is around 2.6 days | Ref. (31), Figure 1, reports a delay of 14 days in the postnatal thymus, while Ref (9) reports a delay between 1.5 and 3.5 days for the fetal thymus |
| The time corresponding to the onset of embryonic thymopoiesis is a parameter of the model ($\text{t}_{0}$) | The estimated value of the time moment when the first progenitor triggers the thymus organogenesis  ~ E10 | In Ref. (14) it is stated that “the thymic rudiment is colonized by hematopoietic precursors by 11 dpc”  In Ref. (13) it is stated that “the first inflow of progenitors has been observed to occur on fetal day 10 to 12” |
| The time corresponding to the appearance of thymocytes of each type are not explicit parameters of the model but are computed based on the estimated parameters | The first DN thymocytes appear around E10.5, the first DP thymocytes appear around E13.5-14 while the SP4 and SP8 cells appear around E14.5-15 | In agreement with experimental results presented in Ref. (13), Figure 2 |

Supplemental Table SII. Validation of the model based on exponentially decreasing proliferation rates (M1.V1), using data from the literature.

**Figure S1**


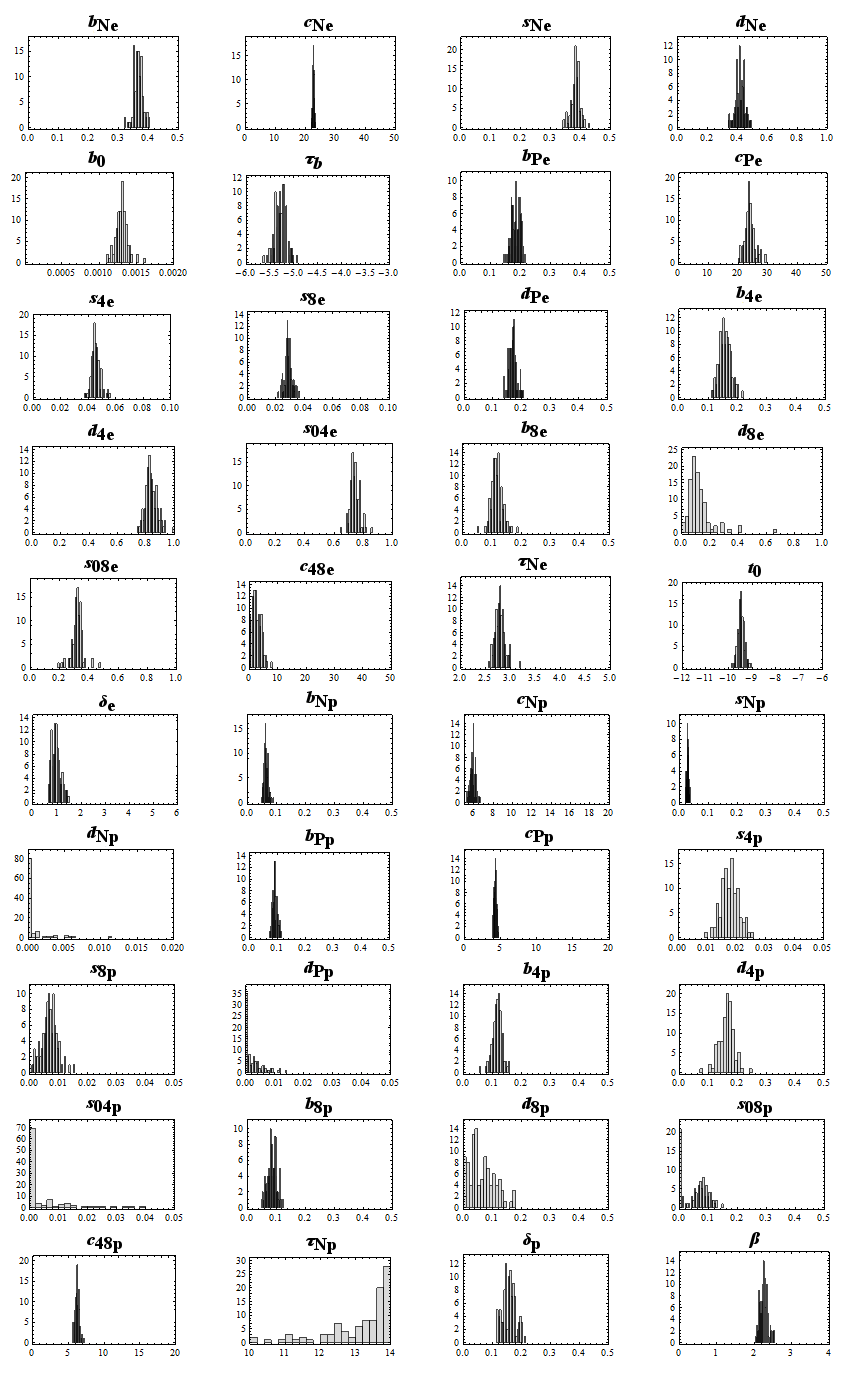


**Supplementary Figure S1.** Histograms of the estimated parameters (parameters containing “e” in their notation correspond to fetal values while those containing “p” corresponds to post-natal values), for the model M1.V1.

**Figure S2**


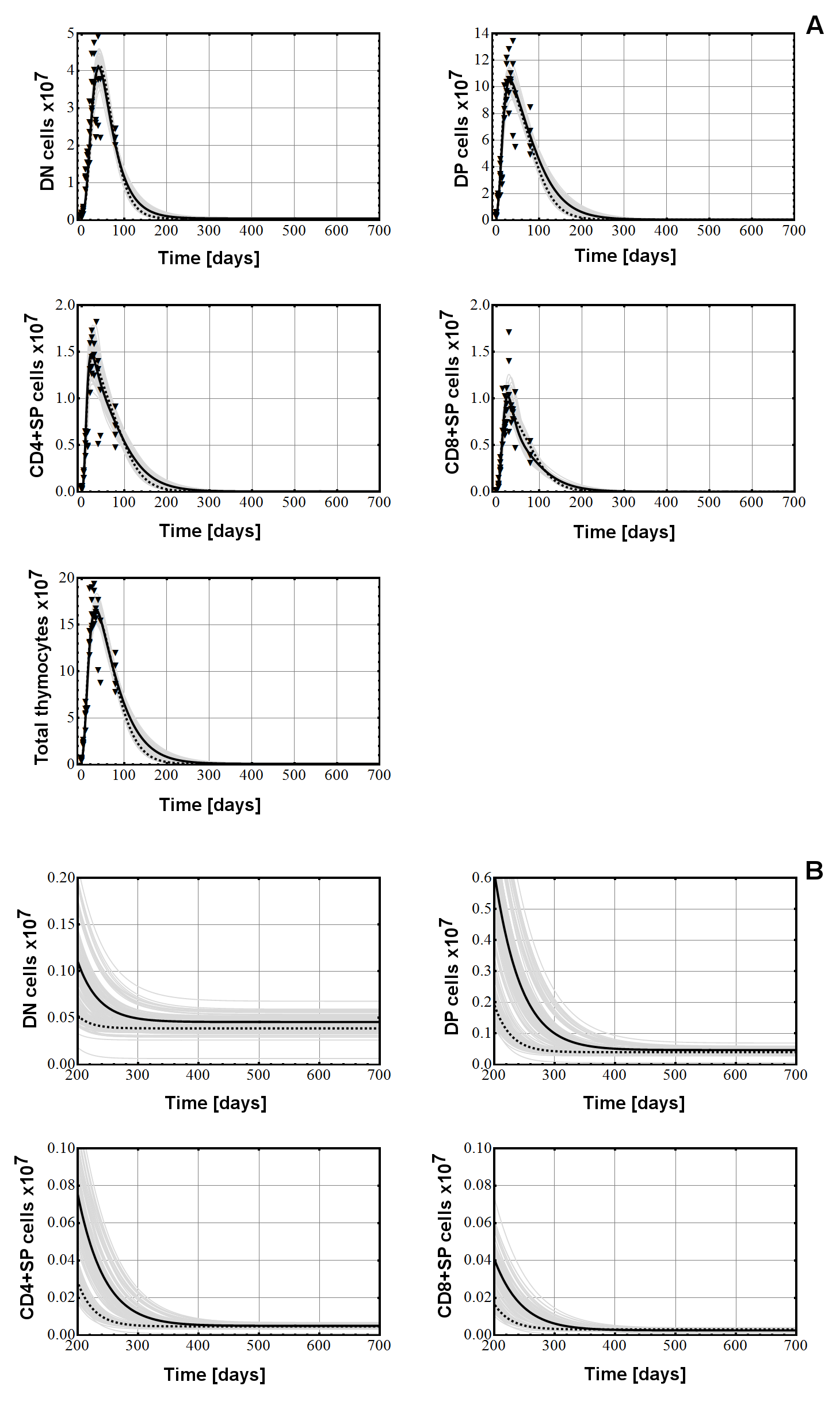


**Supplementary Figure S2.** The dynamics of the four thymocyte populations and the total number of thymocytes from the onset of the fetal thymus development until the death of the animal (A, upper panel), and the dynamics of the four thymocyte populations in the later stages of post-natal life (B, lower panel), corresponding to the two selected variants of the mathematical model: M1.V1 (continuous line), and M2.V1 (dotted line).
